# An integrated model of care for neurological infections: the first six years of referrals to a specialist service at a university teaching hospital in Northwest England

**DOI:** 10.1186/s12879-015-1109-3

**Published:** 2015-09-24

**Authors:** Lance Turtle, Agam Jung, Nick J Beeching, Derek Cocker, Gerry R Davies, Andy Nicolson, Michael BJ Beadsworth, Alastair RO Miller, Tom Solomon

**Affiliations:** Institute of Infection and Global Health, University of Liverpool, Ronald Ross Building, 8 West Derby Street, Liverpool, L69 7BE UK; Tropical and Infectious Disease Unit, Royal Liverpool University Hospital, Prescot Street, Liverpool, L7 8XP UK; NIHR Health Protection Research Unit in Emerging and Zoonotic Infections, Liverpool, L69 7BE UK; Leeds General Infirmary, Leeds, LS1 3EX UK; Clinical Sciences, Liverpool School of Tropical Medicine, Pembroke Place, Liverpool, L3 5QA UK; Walton Centre for Neurology and Neurosurgery NHS Foundation Trust, Liverpool, L9 7LJ UK

## Abstract

**Background:**

A specialist neurological infectious disease service has been run jointly by the departments of infectious disease and neurology at the Royal Liverpool University Hospital since 2005. We sought to describe the referral case mix and outcomes of the first six years of referrals to the service.

**Methods:**

Retrospective service review.

**Results:**

Of 242 adults referred to the service, 231 (95 %) were inpatients. Neurological infections were confirmed in 155 (64 %), indicating a high degree of selection before referral. Viral meningitis (35 cases), bacterial meningitis (33) and encephalitis (22) accounted for 38 % of referrals and 61 % of confirmed neurological infections. Although an infrequent diagnosis (*n* = 19), neurological TB caused the longest admission (median 23, range 5 – 119 days). A proven or probable microbiological diagnosis was found in 100/155 cases (64.5 %). For the whole cohort, altered sensorium, older age and longer hospital stay were associated with poor outcome (death or neurological disability); viral meningitis was associated with good outcome. In multivariate analysis altered sensorium remained significantly associated with poor outcome, adjusted odds ratio 3.04 (95 % confidence interval 1.28 – 7.22, *p* = 0.01).

**Conclusions:**

A service of this type provides important specialist care and a focus for training and clinical research on complex neurological infections.

## Background

Neurological infectious diseases cause an estimated 98.4 years lived with disability per 100 000 people across the globe [1]. Although the majority of this is in developing countries, there is also considerable disease burden in Industrialised Nations [2–4], and the spectrum of patients seen may be changing [5].

In the UK there is often no uniform approach to investigation and management of patients with suspected neurological infections [6–9]. Several questions around the provision of care of neurological infections remain. What is the burden of admissions and length of hospital stay for neurological infectious diseases in a specialist setting? What is the case mix of patients seen in the specialist setting? What are the clinical outcomes? In many British hospitals, such patients are managed by internal medicine but in some centres they are cared for by infectious diseases specialists or neurologists [10]. We established a combined neurological infectious diseases service in 2005 and this report reviews the activity of the first 5 years of this specialist service.

## Methods

We conducted a retrospective review of patients referred to the neurological infectious diseases service between 1^st^ Jan 2005 and 31^st^ December 2009.

The service is run jointly between the Tropical and Infectious Disease Unit of the Royal Liverpool University Hospital (RLUH) and the Walton Centre NHS Foundation Trust (formerly the Walton Centre for Neurology and Neurosurgery). A weekly joint ward round by neurologists and infectious disease physicians is run at the RLUH for inpatients. The service also runs a specialist outpatient clinic. The Tropical and Infectious Diseases Unit (TIDU) currently has responsibility for 32 of the 850 inpatient beds at the Royal Liverpool University hospital and has close clinical links with the Liverpool School of Tropical Medicine and the Institute of Infection and Global Health of the University of Liverpool. The hospital provides secondary care to a population of 750 000, and infectious disease specialist provision is for a wider population of approximately 2 million across the region, including approximately 1500 HIV patients. Inpatient beds on the unit are covered in rotation by four of twelve specialists in infectious diseases and tropical medicine. Quality of care within the service is maintained by action arising from a rolling program of clinical audit of many aspects of patient care; some audits are hospital wide while others apply only to the TIDU. The Walton Centre NHS Foundation Trust is a tertiary care neuroscience centre with 25 full time equivalent consultant neurologists and 14 consultant neurosurgeons. The centre provides care for a population of approximately 3.5 million in a hub and spoke model, with neurologists from the centre visiting all hospitals in the region.

In order to identify patients seen by the service we searched electronic discharge records and clinic letters of the unit for the following terms: encephalitis, meningitis, neuro, neurological infection, neurological, neurologist, CSF; we also reviewed ward admission/discharge books and personal logs kept by individual physicians involved in the service.

Patients who were referred between 1^st^ Jan 2005 and 31^st^ December 2009 were included. Data collected were: age, sex, referral source, length of hospital stay, inpatient or outpatient consultation, presenting syndrome, final diagnosis, microbiological aetiology and outcome. Patients were classified by presenting syndromes (meningism, altered sensorium, focal central nervous system (CNS) signs, peripheral neurological signs, seizures, febrile illness). The patients’ final diagnosis was based on the results of standard microbiological investigations and clinical progress (anti-neuronal antibodies were not included). Patients were categorised into neurological infections and other diagnoses. The presenting clinical features were assessed to see which were predictive of a neurological infection.

Neurological infections were considered proven, probable, possible and unknown according to the following criteria [11]:

- Proven: identification of an organism in the cerebrospinal fluid (CSF) or brain tissue by culture, microscopy, nucleic acid amplification or antigen test.
- Probable: in a patient with an appropriate neurological illness, identification of an organism as above from non-CNS tissue, or identification of a classically implicated organism by immuno-assay (e.g. serology, interferon-gamma release assay), or a well recognised clinical syndrome where diagnostic tests are not normally applied.
- Possible: clinical diagnosis (e.g. scan appearance etc.), non-CNS identification of a non-classical organism, diagnosis by response to treatment.
- Unknown: clinical picture of neurological infection but causative agent unknown.

Statistical analysis was conducted using R version 2.13.1. *P* values of < 0.05 were considered significant. Factors investigated for association with death in hospital or neurological sequelae at discharge were: age, diagnosis, HIV status, altered sensorium at presentation, presence of fungal or parasitic infection, length of hospital stay. For analysis, patients were categorised as having either bad outcome (death or neurological sequelae) or good outcome (full recovery). Analysis of categorical variables was done by Fisher’s exact test, continuous variables by linear regression. Ninety five percent confidence intervals (95 % CI) were calculated. Variables with a *p* < 0.1 were included in multivariate logistic regression models. All patient data were anonymised and stored securely in accordance with current information governance guidelines.

### Ethics statement

The service review was approved by the clinical audit working group of the Royal Liverpool and Broadgreen University Hospitals NHS Trust (ref. 3413–10/11). Formal research and ethics approval was not necessary for a review of patients looked after within our own service.

## Results

Two hundred and forty two patients were referred to the service during the study period (Table 1). Neurological infections were subsequently confirmed in 155 patients (64 %, 149 inpatients and 6 outpatients) and other diagnoses were made in 87 patients. One hundred and thirty four patients (55.4 %) were male, 57 were HIV positive and 174 were HIV negative (ten patients were not HIV tested). Two hundred and thirty one patients were first seen as inpatients and 11 as outpatients. Sixty-nine percent of referrals were from within the hosting hospitals, 17 % were from within 10 miles and 14 % were from more than 10 miles away. There was a trend towards fewer referrals with increasing distance from the service.

**Table 1:** Characteristics of all patients in this study

Number seen for suspected neurological infectious disease	242	
Confirmed neurological infectious disease	138	(57 %)
Neurological complication of an infectious disease	17	(7 %)
No neurological infection or complication	87	(36 %)
Male	134	(55.4 %)
HIV positive	57	(23.6 %)
Median age (years)	39	(IQR 28–52)
Median length of stay (days)	8	(IQR 3–19)
Inpatients	231	
Outpatients	11	
Outcome:		
Full recovery	149	
Sequelae	53	
Death	9	
Missing data (though alive at discharge)	31	

### Presenting syndromes

There was a wide spectrum of presenting problems (Table 2). The classic triad of fever, headache and abnormal neurology (seizures, altered sensorium or focal neurological signs) was present in only eight patients (all of whom had confirmed neurological infections). Many patients had features of meningism and photophobia without focal neurological signs. Meningism and altered sensorium were significantly associated with neurological infections (meningism OR 2.06, 95 % CI 1.09–4.03; altered sensorium OR 2.68, 95 % CI 1.36–5.53). Headache was more frequent in patients who went on to have neurological infection ruled out.

**Table 2:** Clinical presentation of 242 patients with suspected neurological infectious disease, divided according to confirmed neurological infection or other diagnosis

	Neurological infectious disease (*n* = 150)	Other diagnosis (*n* = 91)	*p* value
Fever	33 (22 %)	24 (26 %)	0.44
Headache	45 (30 %)	42 (46 %)	0.01
Meningism	53 (35 %)	19 (21 %)	0.02
Altered sensorium	52 (35 %)	15 (16 %)	0.002
Seizures	13 (9 %)	14 (15 %)	0.14
Focal deficit	11 (7 %)	9 (10 %)	0.48
Peripheral nerve lesion	10 (7 %)	9 (10 %)	0.46
Fever + abnormal neurological finding ^a^	23 (15 %)	6 (7 %)	0.06
Headache + abnormal neurological finding ^a^	16 (11 %)	7 (8 %)	0.51
Fever + headache + abnormal neurological finding ^a^	8 (5 %)	0	0.026

Data represent the number of patients for each individual clinical finding, and patients may be counted more than once

^a^ Abnormal neurological finding is any of: altered sensorium, seizures or focal neurological signs. These groupings count each individual patient once only

### Final diagnoses

**Fig. 1:**
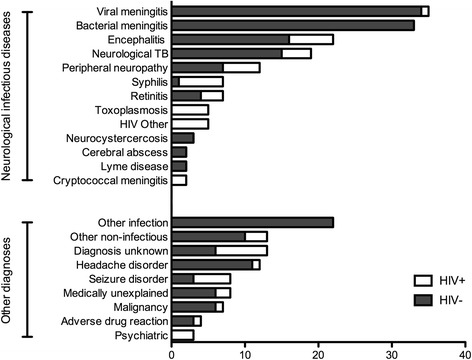
Final diagnoses made in 242 patients referred with suspected neurological infection. “HIV other” refers to two cases of HIV associated myelopathy, one case of optic neuropathy, one adverse drug reaction to anti-retroviral therapy, one case of cerebral vasculitis and one with Guillain Barré syndrome (the presenting syndrome of HIV infection)

Viral meningitis, bacterial meningitis and encephalitis were the most common diagnoses accounting for 61 % of all neurological infections and 38 % of all 242 patients in the study (Fig. 1). Other, non-neurological, infections were mostly patients with non-specific febrile illness but included two cases of non-neurological TB, two of sinusitis, and one each of legionnaires disease, tonsillitis, *Staphylococcus aureus* and *Proteus* spp. bacteraemia. Other non-infectious conditions included Parkinson’s disease, benign intracranial hypertension, hypertensive encephalopathy, transverse myelitis, sarcoidosis, vasculitis and stroke.

The diagnoses made in HIV positive patients were different to those in the HIV negative patients (Fig. 1); in particular there were fewer cases of meningitis. Two cases of cryptococcal meningitis occurred only in HIV positive patients. Toxoplasmosis, syphilis, seizure disorders, psychiatric diagnoses and unknown diagnoses were all more common in the HIV positive patients (Fig. 1). All the patients with suspected TBM had a final diagnosis of tuberculosis; in addition three other patients had non-meningitic neurological TB, two had space occupying lesions (SOL) and one had choroidal TB.

### Causative organisms and diagnostic certainty

The organisms implicated in the patients diagnosed with neurological infection are shown in Table 3. In all, 100 of 155 patients (64.5 %) had a proven or probable microbiological diagnosis. A possible aetiological diagnosis was made in 20 patients (13 %) and for 34 patients (22 %) the causative organism was unknown. Bacterial infection was the most common (58 cases, including tuberculosis), followed by viral (50 cases) and lastly fungal/parasitic (11 cases).

**Table 3:** Breakdown of microbiological aetiology and certainty of diagnosis

Diagnosis	Organism	Certainty		
		Proven	Probable	Possible
Viral meningitis	Total	14	1	2
	*Herpes simplex virus 2*	7	0	0
	*Enterovirus*	5	0	0
	*Varicella zoster virus*	1	0	0
	*Epstein Barr virus*	1	0	0
	*Mumps* * virus*	0	1	2
Bacterial meningitis	Total	23	5	0
	*Streptococcus pneumoniae*	7	4	0
	*Neisseria meningitidis*	8	1	0
	*Listeria monocytogenes*	3	0	0
	*Pseudomonas aeruginosa*	1	0	0
	*Streptococcus pyogenes*	1	0	0
	*Haemophilus influenzae*	1	0	0
	*Eschericia coli*	1	0	0
	Mixed	1	0	0
Encephalitis	Total	8	6	1
	*Herpes simplex virus 1*	6	0	1
	*Cytomegalovirus*	1	0	0
	*Human herpes virus 6*	1	0	0
	*Varicella zoster virus*	0	1	0
	HIV	0	5	0
TBM	*Mycobacterium tuberculosis*	4	2	13
Peripheral neuropathy	Total	0	7	0
	*Herpes simplex virus 1&2*	0	2	0
	*Cytomegalovirus*	0	1	0
	*Varicella zoster virus*	0	1	0
	*Salmonella typhi*	0	1	0
	HIV	0	3	0
Retinitis	Total	4	3	0
	*Cytomegalovirus*	2	3	0
	*Varicella zoster virus*	2	0	0
Cerebral abscess		2	0	0
	*Aspergillus* sp.	1	0	0
	*Nocardia* sp.	1	0	0
Other syndromes				
Syphilis	*Treponema pallidum*	0	5	2
Toxoplasmosis	*Toxoplasma gondii*	0	5	0
Cysticercosis	*Taenia solium*	0	1	2
Crytococcal meningitis	*Cryptococcus neoformans*	1	1	0
Lyme disease	*Borrelia burgdoferi*	0	2	0
Optic neuropathy	HIV	0	1	0
Myelopathy	HIV	0	2	0
Cerebral vasculitis	HIV	0	1	0
Antiretroviral drug reaction	HIV	0	1	0
Stroke, septic embolus	Group C *Streptococcus*	1	0	0
Grand Totals		57	44	20

The table shows data for 121 patients for whom a microbiological diagnosis was confirmed. A further 34 patients were diagnosed as having a neurological infectious disease, but the causative organism was unknown

### Outcome

Out of 242 patients, 149 recovered completely, 53 recovered with neurological sequlae and nine died. For 31 patients their neurological condition was not clear from the records, though they were alive at hospital discharge. Full recovery occurred most often after viral meningitis and least often after encephalitis or neurological TB. Of the nine patients who died, eight had neurological infections, comprising TBM (three), toxoplasmosis (two), bacterial meningitis (one *Listeria monocytogenes* and one *Escherichia coli*) and cytomegalovirus (CMV) encephalitis; the ninth patient was HIV positive and had cerebral lymphoma. The median age of patients who died was 49.5 years (interquartile range (IQR) 28 to 52.25) and the median age of patients who survived was 41 (IQR 28 to 56).

### Factors associated with poor outcome

In patients with confirmed neurological infections, altered sensorium, age and longer hospital stay were associated with poor outcome; viral meningitis was associated with good outcome (Table 4). In a multivariate logistic regression model only altered sensorium at presentation was significantly associated with poor outcome (Table 4). Data on outcome were missing for 25 patients; sensitivity analysis showed they could not significantly affect the analysis unless all of the patients with altered sensorium and missing data had complete recovery.

**Table 4:** Odds ratios for risk of poor outcome (death or neurological sequelae at discharge) inpatients with neurological infectious diseases

**Individual variables**	**OR**	**95 % CI**	***p*** **value**
Altered sensorium	4.66	2.02 – 11.1	< 0.001
Viral meningitis	0.08	0.009 – 0.35	< 0.001
Age	1.04	1.02 – 1.06	< 0.001
Length of stay	1.03	1.01 – 1.05	< 0.001
**Multivariate** **analysis**	**AOR**	**95 % CI**	***p>*** **value**
Altered sensorium	3.04	1.28 – 7.22	0.01
Viral meningitis	0	NA	< 0.001
Age	1.02	0.99 – 1.05	0.14
Length of admission	1.01	1 – 1.03	0.08

*OR*, odds ratio, *AOR*, adjusted odds ratio

### Workload of the service

The 241 patients occupied 2841 bed days over a five-year period, median length of stay in hospital (LOS) was 9 days (IQR 4 – 20 days) compared with 5 days for other patients. Thus the 241 patients represented 5.9 % of the 4057 admissions to the TIDU over the 5 years, but accounted for 8.5 % of all bed days. Median LOS for viral meningitis was 4 days (IQR 2–6 days), significantly shorter than for patients with encephalitis (LOS 18 days IQR 13–32 days), bacterial meningitis (LOS 16 days, IQR 9–21 days) and TBM (LOS 23 days, IQR 16–50 days) (*p* < 0.0001). Twelve out of 19 patients with TBM (63 %) were in the 4^th^ quartile of LOS.

## Discussion

This study represents the experience of a specialist neurological infections service in the UK, seeing 242 patients over five years. We know of few such services; one department in North America has reported the features of 116 referrals over a 15 month period [4]. This equates to 1.8 new patients per week as opposed to approximately 1 per week in our study.

In our previous broader survey of clinical practice in ten district general hospitals in the region, 385 patients with suspected neurological infection were seen over a three months, just over 3 patients per week in each hospital [10]. In our specialist service neurological infections were confirmed in 62 % of patients, compared with 11 % in the regional study [10]. These differences are likely due to the fact that only patients with highly suspected diagnoses are referred by other medical teams to the specialist service. In common with other case series of this type, viral meningitis was the most common diagnosis in our study.

A minority of patients in this study exhibited the classic presentation of fever, headache and focal neurological signs or meningism, yet many had neurological infections diagnosed. This is consistent with other studies showing 10 % of all neurological infections and 44 % of bacterial meningitis cases presenting with the classic features of these conditions [4, 12]. This suggests that the “classic presentation” of a neurological infection may not be the rule. Many patients in this study presented with photophobia and were labelled as having meningism but received final diagnoses other than a neurological infection. Photophobia is a subjective feature of meningism that is at risk of being incorrectly labelled.

A definite or probable microbiological diagnosis was reached in 64.5 % of our patients. This figure is relatively high compared with other published reports conducted in a similar setting [2, 3] and is to be expected as the service sees selected patients for whom the prior probability of a neurological infection is high. Moreover, those studies focussed on encephalitis where a definitive diagnosis is more difficult to reach [2, 3]. A weakness of our study is that autoimmune encephalopathies were not tested for, and some cases may have been missed.

Despite the small numbers of deaths in this study, outcomes compare favourably with those in other reports, especially considering the referral bias towards more severe cases [12–14]. In our study the association of young age and short length of stay with good outcome are likely proxy markers of viral meningitis. Although we have not specifically addressed this question, several other studies have suggested that specialist infection services raise significant new differential diagnoses [15] and improve diagnostic efforts [16], and outcome [17]. It is likely that a service as described here has the potential to significantly improve outcome.

Fifty seven (23.6 %) of the patients were HIV positive, with similar median age of 39 (range 25–68) years compared to those without HIV (40, range 17–94). Overall, a neurological presentation was the first manifestation of HIV in 12 of these 57 patients, similar to our previous description of neurological features in 29/176 (16.5 %) adults at first presentation with HIV [18]. Five patients with HIV infection had HIV encephalitis/dementia, which is a recognised seroconversion illness as well as a late feature of HIV [18, 19]. There were some unusual diagnoses in HIV positive patients, including optic atrophy, cerebral vasculitis and atypical Guillian-Barré syndrome which have all been associated with HIV infection [20–22]. Psychiatric problems are common in HIV positive patients, but neurological infectious diseases are also common and can have severe consequences [23]. It is therefore not surprising that the threshold for investigation for CNS infection in HIV positive patients is low and is prompted by any change in mental status, resulting in a number of psychiatric diagnoses in this cohort.

In addition, 7 patients (6 HIV positive) had neurological syphilis, emphasizing the need to consider testing for syphilis as well as for HIV in all patients with possible neurological infection, especially anyone with dementia, meningitis or uveitis [24].

This study is limited by its retrospective nature and it is likely that some patients referred to the service have not been included in this report. Similarly, some patients may have not been referred to the service but managed by general physicians. To try and overcome this, we searched ICD-10 coded discharge data from our institution. Unfortunately these data were not sufficiently accurate for inclusion in the study. However, the number of episodes identified was small, making it unlikely that we have missed enough patients to affect our conclusions.

Only nine deaths occurred in the study cohort, making it difficult to reach conclusions on the factors predictive of death. A further weakness of our study is that we did not collect data on time to treatment, another variable that can affect outcome of neurological infections [25–28].

## Conclusions

This study found that approximately one patient per week was referred to a specialist neurological infection service. The patients with confirmed neurological infections alone accounted for 8.5 % of total bed occupancy on the unit although they comprised only 5.6 % of admissions. Therefore, although the numbers of patients are relatively small, neurological infectious diseases have a disproportionately high impact on service usage. The overall mortality of confirmed neurological infections was relatively low at 5 %, with mortality highest in those with altered sensorium. This service also provides a focus for clinical training and research to improve our understanding of these relatively uncommon but serious infections.

## Abbreviations

AOR: Adjusted odds ratio
CI: Confidence interval
CMV: Cytomegalovirus
CNS: Central nervous system
CSF: Cerebrospinal fluid
HIV: Human immunodeficiency virus
HSV: Herpes simplex virus
IQR: Interquartile range
OR: Odds ratio
NHS: National Health Service
LOS: Length of stay
RLUH: Royal Liverpool University Hospital
SOL: Space occupying lesion
sp: Species
TB: Tuberculosis
TBM: Tuberculous meningitis

## References

[1] Dua T, Janca A, Kale R, Montero F, Mudcetta A, Peden M. Global Burden of Neurological Disorders: estimations and projections. In: Aarli JA, Avanzini G, Bertolote JM, de Boer H, Breivik H, Dua T, et al., editors. Geneva: World Health Organisation; 2007. pp. 1–14. Available from: http://www.who.int/entity/mental_health/neurology/chapter_2_neuro_disorders_public_h_challenges.pdf.

[2] Glaser CA, Honarmand S, Anderson LJ, Schnurr DP, Forghani B, Cossen CK (2006). Beyond viruses: clinical profiles and etiologies associated with encephalitis. Clin Infect Dis.

[3] Granerod J, Ambrose HE, Davies NW, Clewley JP, Walsh AL, Morgan D (2010). Causes of encephalitis and differences in their clinical presentations in England: a multicentre, population-based prospective study. Lancet Infect Dis.

[4] Tan K, Patel S, Gandhi N, Chow F, Rumbaugh J, Nath A (2008). Burden of neuroinfectious diseases on the neurology service in a tertiary care center. Neurology.

[5] Zunt JR (2002). Central nervous system infection during immunosuppression. Neurol Clin.

[6] Michael B, Menezes BF, Cunniffe J, Miller A, Kneen R, Francis G (2010). Effect of delayed lumbar punctures on the diagnosis of acute bacterial meningitis in adults. Emerg Med J.

[7] Bell DJ, Suckling R, Rothburn MM, Blanchard T, Stoeter D, Michael B (2009). Management of suspected herpes simplex virus encephalitis in adults in a U.K. teaching hospital. Clin Med.

[8] Kneen R, Solomon T, Appleton R (2002). The role of lumbar puncture in suspected CNS infection--a disappearing skill?. Arch Dis Child.

[9] Solomon T, Hart IJ, Beeching NJ (2007). Viral encephalitis: a clinician’s guide. Pract Neurol.

[10] Michael BD, Sidhu M, Stoeter D, Roberts M, Beeching NJ, Bonington A (2010). Acute central nervous system infections in adults--a retrospective cohort study in the NHS North West region. QJM.

[11] Granerod J, Cunningham R, Zuckerman M, Mutton K, Davies NWS, Walsh AL (2010). Causality in acute encephalitis: defining aetiologies. Epidemiol Infect.

[12] van de Beek D, de Gans J, Spanjaard L, Weisfelt M, Reitsma JB, Vermeulen M (2004). Clinical features and prognostic factors in adults with bacterial meningitis. N Engl J Med.

[13] Durand ML, Calderwood SB, Weber DJ, Miller SI, Southwick FS, Caviness VS (1993). Acute bacterial meningitis in adults. A review of 493 episodes. N Engl J Med.

[14] Solomon T, Michael BD, Smith PE, Sanderson F, Davies NWS, Hart IJ (2012). Management of suspected viral encephalitis in adults--Association of British Neurologists and British Infection Association National Guidelines. J Infect.

[15] Vehreschild JJ, Morgen G, Cornely OA, Hartmann P, Koch S, Kalka-Moll W (2013). Evaluation of an infectious disease consultation programme in a German tertiary care hospital. Infection.

[16] Borer A, Gilad J, Meydan N, Schlaeffer P, Riesenberg K, Schlaeffer F (2004). Impact of regular attendance by infectious disease specialists on the management of hospitalised adults with community-acquired febrile syndromes. Clin Microbiol Infect.

[17] Schmitt S, McQuillen DP, Nahass R, Martinelli L, Rubin M, Schwebke K (2013). Infectious diseases specialty intervention is associated with decreased mortality and lower healthcare costs. Clin Infect Dis.

[18] Ratcliffe L, Thomas S, Beeching NJ, Phillips-Howard PA, Taegtmeyer M (2011). Acute presentations of HIV are still missed in low prevalence areas. Postgrad Med J.

[19] Newton PJ, Newsholme W, Brink NS, Manji H, Williams IG, Miller RF (2002). Acute meningoencephalitis and meningitis due to primary HIV infection. BMJ.

[20] Larsen M, Toft PB, Bernhard P, Herning M (1998). Bilateral optic neuritis in acute human immunodeficiency virus infection. Acta Ophthalmol Scand.

[21] Saravanan M, Turnbull IW (2009). Brain: non-infective and non-neoplastic manifestations of HIV. Br J Radiol.

[22] Sloan DJ, Nicolson A, Miller ARO, Beeching NJ, Beadsworth MBJ (2008). Human immunodeficiency virus seroconversion presenting with acute inflammatory demyelinating polyneuropathy: a case report. J Med Case Rep.

[23] Skiest DJ (2002). Focal neurological disease in patients with acquired immunodeficiency syndrome. Clin Infect Dis.

[24] González-Duarte A, López ZM (2013). Neurological findings in early syphilis: a comparison between HIV positive and negative patients. Neurol Int.

[25] Proulx N, Frechette D, Toye B, Chan J, Kravcik S (2005). Delays in the administration of antibiotics are associated with mortality from adult acute bacterial meningitis. QJM.

[26] Auburtin M, Wolff M, Charpentier J, Varon E, Le Tulzo Y, Girault C (2006). Detrimental role of delayed antibiotic administration and penicillin-nonsusceptible strains in adult intensive care unit patients with pneumococcal meningitis: the PNEUMOREA prospective multicenter study. Crit Care Med.

[27] Raschilas F, Wolff M, Delatour F, Chaffaut C, De Broucker T, Chevret S (2002). Outcome of and prognostic factors for herpes simplex encephalitis in adult patients: results of a multicenter study. Clin Infect Dis.

[28] Køster-Rasmussen R, Korshin A, Meyer CN (2008). Antibiotic treatment delay and outcome in acute bacterial meningitis. J Infect.

